# Diagnostic Accuracy of Artificial Intelligence (AI) to Detect Early Neoplasia in Barrett's Esophagus: A Non-comparative Systematic Review and Meta-Analysis

**DOI:** 10.3389/fmed.2022.890720

**Published:** 2022-06-22

**Authors:** Jin Lin Tan, Mohamed Asif Chinnaratha, Richard Woodman, Rory Martin, Hsiang-Ting Chen, Gustavo Carneiro, Rajvinder Singh

**Affiliations:** ^1^Department of Gastroenterology and Hepatology, Lyell McEwin Hospital, SA Health, Elizabeth Vale, SA, Australia; ^2^Faculty of Health and Medical Sciences, The University of Adelaide, Adelaide, SA, Australia; ^3^Flinders Centre for Epidemiology and Biostatistics, College of Medicine and Public Health, Flinders University, Bedford Park, SA, Australia; ^4^Australian Institute for Machine Learning, The University of Adelaide, Adelaide, SA, Australia

**Keywords:** Barrett's esophagus, dysplasia, esophageal adenocarcinoma, artificial intelligence, deep learning

## Abstract

**Background and Aims:**

Artificial Intelligence (AI) is rapidly evolving in gastrointestinal (GI) endoscopy. We undertook a systematic review and meta-analysis to assess the performance of AI at detecting early Barrett's neoplasia.

**Methods:**

We searched Medline, EMBASE and Cochrane Central Register of controlled trials database from inception to the 28th Jan 2022 to identify studies on the detection of early Barrett's neoplasia using AI. Study quality was assessed using Quality Assessment of Diagnostic Accuracy Studies – 2 (QUADAS-2). A random-effects model was used to calculate pooled sensitivity, specificity, and diagnostics odds ratio (DOR). Forest plots and a summary of the receiving operating characteristics (SROC) curves displayed the outcomes. Heterogeneity was determined by *I*^2^, Tau^2^ statistics and *p*-value. The funnel plots and Deek's test were used to assess publication bias.

**Results:**

Twelve studies comprising of 1,361 patients (utilizing 532,328 images on which the various AI models were trained) were used. The SROC was 0.94 (95% CI: 0.92–0.96). Pooled sensitivity, specificity and diagnostic odds ratio were 90.3% (95% CI: 87.1–92.7%), 84.4% (95% CI: 80.2–87.9%) and 48.1 (95% CI: 28.4–81.5), respectively. Subgroup analysis of AI models trained only on white light endoscopy was similar with pooled sensitivity and specificity of 91.2% (95% CI: 85.7–94.7%) and 85.1% (95% CI: 81.6%−88.1%), respectively.

**Conclusions:**

AI is highly accurate at detecting early Barrett's neoplasia and validated for patients with at least high-grade dysplasia and above. Further well-designed prospective randomized controlled studies of all histopathological subtypes of early Barrett's neoplasia are needed to confirm these findings further.

## Introduction

Artificial Intelligence (AI) is rapidly evolving in gastrointestinal (GI) endoscopy. This development has been promising in the lower gastrointestinal tract, where various AI-assisted algorithms to detect and diagnose colorectal lesions during colonoscopy have been utilized (1). There are fewer studies on detecting early neoplasia associated with Barrett's esophagus (BE) (1). BE is a metaplastic alteration of the normal esophageal epithelium detected on endoscopic examination and pathologically confirmed as exhibiting intestinal metaplasia. It is considered a precursor to the development of esophageal adenocarcinoma, which carries high mortality (2). According to the Surveillance, Epidemiology, and End Results Program (SEER) database, the incidence of esophageal carcinoma is rising more rapidly than any other form of cancer, with a 6-fold increase demonstrated from 1975 to 2001(3). The overall 5-year survival rate for patients with esophageal adenocarcinoma in the United States is a dismal 21% (4).

Studies have shown that patients in whom adenocarcinoma is detected during endoscopic surveillance for BE are more likely to have early Barret's esophageal cancer, receive curative therapy, and survive longer than symptomatic patients in whom adenocarcinoma is detected (5). Surveillance strategies utilizing white light endoscopy (WLE) and random biopsies have been advocated and critical in recognizing dysplasia. However, dysplastic lesions are still difficult to distinguish from non-dysplastic mucosa as only a tiny fraction of Barrett's esophagus mucosa is randomly biopsied. It is difficult to obtain an accurate assessment of the presence of cancer or dysplasia due to sampling error (6). It has been shown that surveillance endoscopy done at non-expert centers has a lower early BE cancer detection rate (7). Expertise and advanced techniques such as Narrow Band Imaging (NBI) and magnification can improve accuracy in diagnosing early BE cancer but are only available in expert centers (8). Better and more widespread techniques to enhance the accuracy of endoscopic surveillance of early BE cancer is required.

Robust evidence was lacking in previous meta-analyses to support the use of AI in the surveillance of early Barrett's neoplasia, as most included studies were retrospective in nature (1, 9, 10). Moreover, these meta-analyses reviewed the performance of AI in the upper gastro-intestinal tracts of various other pathologies with very few studies on early Barrett's neoplasia. In addition, there is a lack of ground truth on whether images had correlated with actual histopathological reports. While the meta-analyses reported the generic class of AI algorithm used in included studies, the specific type of algorithm used, which is an important consideration for its overall accuracy, were not reported. We provide an updated study aiming to collate ongoing evidence on recently published studies on the utility of AI in detecting dysplasia and early cancer in BE.

## Methods

This study was pre-registered with the PROSPERO register (11) and followed the Preferred Reporting Items for Systematic Reviews and Meta-Analyses of Diagnostic Test Accuracy (PRISMA-DTA) (12).

### Eligibility Criteria

The inclusion criteria utilized the PICO methodology and included:

- **P**articipants: Patients with Barrett's esophagus with or without dysplasia;
- **I**ntervention: Use of artificial intelligence or computer-assisted diagnosis for detection of early Barrett's neoplasia;
- **C**ontrol: Standard surveillance of Barrett's esophagus with White Light Endoscopy (WLE) with or without Narrow Band Imaging (NBI);
- **O**utcome measures the accuracy, sensitivity, specificity, area under the receiver operating characteristic (AUROC) curves of AI models to detect early Barrett's neoplasia.

The exclusion criteria were as follows: (i) Endoscopic surveillance technique such as volumetric laser endomicroscopy and hyperspectral imaging; (ii) Histological subtypes such as squamous cell carcinoma (iii) Reviews, meta-analyses, editorials, letters, comments (iv) and animal studies.

### Search Strategy

An electronic search was performed by Medline, EMBASE and Cochrane Central Register of controlled trials database from inception to the 28th Jan 2022 using the following MeSH terms or free text: “artificial intelligence,” “AI,” “convolutional neural network,” “deep learning,” “computer-assisted diagnosis,” “computer-aided detection,” “Barrett's esophagus,” “dysplasia,” “adenocarcinoma,” “esophageal adenocarcinoma” and “esophageal tumor” (Supplementary Tables 1–3). The search was limited to human studies, but there were no language restrictions. Two independent reviewers (J.T and R.M) performed an initial literature search and selected relevant studies based on the eligibility criteria. Titles and abstracts were screened to exclude studies that did not address the research questions. Subsequently, the remaining studies were assessed in full for eligibility. Finally, any discrepancies were resolved by consensus between the two reviewers or discussion with a third senior author (M.A.C).

### Data Collection and Study Quality Assessment

The following data were extracted from each study: author, year, journal, country or region, types of study, endoscopic imaging modality used, details of artificial intelligence algorithm used, definitions of Barrett's dysplasia, types of controls used, whether images or videos had confirmed corresponding histology, number of patients, endoscopic images or videos, rates of true positivity, false negativity, true negativity, false positivity, sensitivity, specificity, accuracy or area under receiver operating characteristics curve of respective artificial intelligence algorithm used in each study, and whether the study had been validated internally or externally, or achieved a real-time diagnosis. Two investigators (J.T and R.M) extracted the data independently. Study quality was assessed using Quality Assessment of Diagnostic Accuracy Studies – 2 (QUADAS-2) by two independent reviewers (J.T and R.M) (13). Conflicts were resolved by discussion and involvement of a third senior author (M.A.C). QUADAS-2 contains four key domains: patient selection, index test, reference standard, and flow of patients through the study. Each domain is assessed in terms of risk of bias, and the first three are also evaluated in terms of concerns regarding applicability. Risk of bias and applicability were both judged as “low,” “high,” or “unclear”.

### Statistical Analysis

A random-effects model, as described by DerSimonian and Laird, was used to calculate the following: pooled sensitivity, specificity and diagnostics odds ratio (DOR). Forest plots and a summary of the receiver operating characteristics (SROC) curves were used to display the outcomes. The SROC plot summarizes the sensitivity and specificity of individual studies on a scatter plot, together with the summary operating point to depict the overall accuracy.

*I*^2^, Tau^2^ statistics and *p*-value were used to assess heterogeneity. An *I*^2^ of >50%, Tau^2^ of >0.1 or *p* < 0.05 implies significant heterogeneity. Publication bias was visually assessed using funnel plots and Deek's funnel plot asymmetry test (*p* < 0.05 implying publication bias). Forest plots were performed using the meta package in R Project for Statistical Computing, developed by the R Foundation (14), and all other statistical analyses were performed using the MIDAS package in STATA Statistical Software (15).

## Results

### Search Results

**Figure 1:**
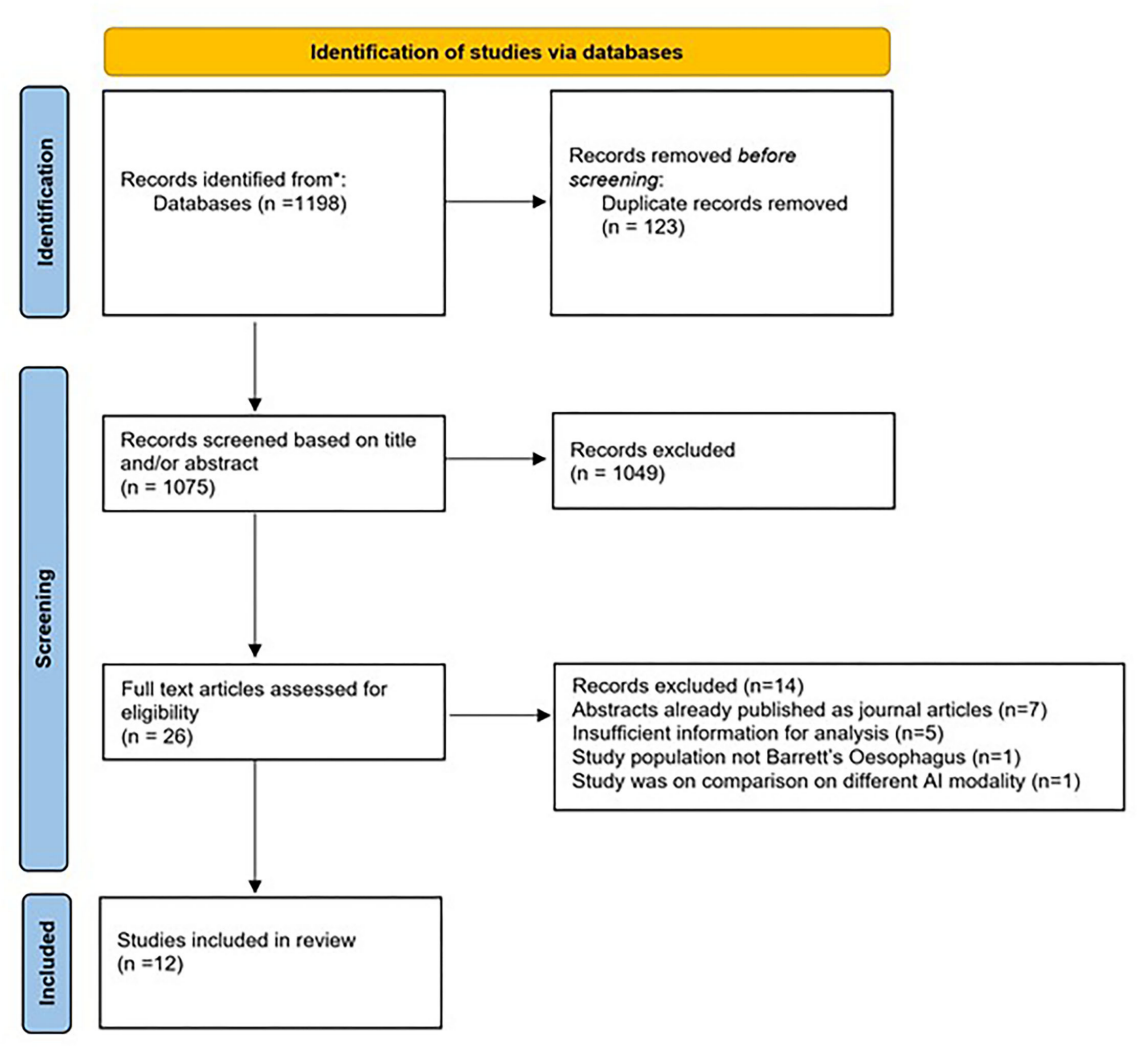
Modified PRISMA flow diagram of the search strategy and study selection.

The initial search strategy yielded 1,198 studies. Following title and abstract screening, 1,172 articles were excluded. The remaining twenty-six studies were reviewed in full text. Fourteen studies were further excluded as they did not meet the eligibility criteria. Finally, twelve studies that met all the inclusion criteria were included for this meta-analysis (16–27). The search strategy and study selection are shown in Figure 1.

### Study Characteristics

Table 1 illustrates the study characteristics of included studies. A total of 1,361 patients were included in these studies, with 532,328 images acquired to train the various AI models that were developed. Seven studies were retrospective (16, 17, 19, 22, 24, 26, 27), four studies were prospective (18, 21, 23, 25), and one study had both retrospective and prospective components in their methods (20). In terms of endoscopic imaging modality evaluated, eleven studies included WLE (16–26), three studies included NBI (17, 22, 27), and three studies included virtual chromoendoscopy images (i-Scan) (24–26). More recent studies from 2019 used Convolutional Neural Networks (CNN) instead of Support Vector Machine (SVM) as the basis of their AI model (17, 19–27). Three studies had used a proprietary pre-training dataset of images from various parts of the gastrointestinal tracts known as “GastroNet” (20, 21, 27). Only two studies demonstrated the real-time capabilities of their AI models (21, 22). Eleven studies documented that endoscopic images included were confirmed on histopathological reports (16–22, 24–27). Among all included studies, there were slight differences in the definitions of dysplastic lesions, including endoscopic images of BE with high-grade dysplasia, T1/intra-mucosal adenocarcinoma, esophageal adenocarcinoma, or a combination of the above. Based on the QUADAS-2 scale, seven studies had low risks of bias, four studies had unclear risks of bias, and one study contained risks of bias (Table 2). There were no concerns regarding the applicability of all included studies.

**Table 1:** Study characteristics of included studies.

**Author**	**Year**	**Region** **(Country)**	**Study type**	**Imaging** **modality**	**AI** **Type**	**Pre-** **training**	**CNN** **model**	**Real** **time**	**Dysplasia** **inclusion**	**Histology** **confirmed**	**Training** **images (n)**	**Patients**	**Sen**	**Spec**
Van Der Sommen (16)	2016	Netherlands	Retrospective	WLE	SVM	NA	NA	N	HGD/EAC	Y	100	44	83%	83%
Ebigbo (17)	2019	Germany	Retrospective	WLE	CNN	ImageNet	ResNet	N	EAC(pT1)	Y	148	62	97%	88%
				NBI									94%	80%
				WLE							100	39	92%	100%
de Groof (18)	2019	Europe (Netherlands, Germany, Belgium)	Prospective	WLE	SVM	NA	NA	N	HGD/EAC	Y	60	60	95%	85%
Abdelrahim (19)	2020	UK	Retrospective	WLE	CNN	NM	SegNet	N	NM	Y	251	NM	93%	78%
de Groof (1) (20)	2020	Europe (Netherlands, France, Belgium)	Retrospective	WLE	CNN	GastroNet	ResNet-Unet	N	HGD/EAC	Y	1,247	414	87.6%	88.6%
			Retrospective								1,544	509	90.0%	87.5%
			Prospective								1544	509	92.5%	82.5%
de Groof (2) (21)	2020	Netherlands	Prospective	WLE	CNN	GastroNet	ResNet-Unet	Y	HGD/EAC	Y	1,544	509	76%	86%
Hashimoto (22)	2020	USA	Retrospective	WLE/ NBI	CNN	ImageNet	Inception- ResNetv2	Y	HGD/T1	Y	1,832	100	96.4%	94.2%
Samarasena (23)	2021	USA	Prospective	WLE	CNN	ImageNet	Xception	N	HGD/T1	Y	4,000	150	95%*	97.6%*
Hussein (1) (24)	2021	Europe (UK, Spain, Belgium)	Retrospective	WLE/ i-Scan	CNN	NM	ResNet 101	N	HDG/ IAC	Y	76,496	58	82%	82%
Hussein (2) (25)	2021	Europe (UK, Spain, Belgium)	Prospective	WLE/ i-Scan	CNN	NM	ResNet 101	N	HDG/ IAC	Y	26,6930	65	88.3%	80.1%
Hussein (3) (26)	2021	Europe (UK, Spain, Belgium)	Retrospective	WLE/i-Scan	CNN	NM	FCN ResNet 50	N	HDG/ IAC	Y	14,8936	124	90.5%	80.4%
Struyvenberg (27)	2021	Europe (Netherlands, Sweden, Germany)	Retrospective	NBI	CNN	GastroNet	ResNet-Unet	N	HGD/ EAC	Y	183	100	88%	78%
											30,204	150	75%*	90%*

*WLE, white light endoscopy; NBI, narrow band imaging; CNN, convolutional neural networks; SVM, support vector machine; NA, Not applicable; NM, Not Mentioned;*

* *refers to performance of video validation; HGD, high grade dysplasia; EAC, early adenocarcinoma; IAC, intramucosal adenocarcinoma; Sen, sensitivity, Spec, specificity*.

**Table 2:** Quality assessment of diagnostic accuracy studies – 2 of included studies.

**Study**	**Risk of bias**				**Applicability concerns**		
	**Patient selection**	**Index test**	**Reference standard**	**Flow and timing**	**Patient selection**	**Index test**	**Reference standard**
Van Der Sommen (16)							
Ebigbo (17)							
de Groof (18)							
Abdelrahim (19)							
de Groof (1) (20)							
de Groof (2) (21)							
Hashimoto (22)							
Samarasena (23)							
Hussein (1) (24)							
Hussein (2) (25)							
Hussein (3) (26)							
Struyvenberg (27)							

*Green, low risk; Orange, unclear risk; Red, high risk*.

### AI and Detection of Early BE Neoplasia

**Figure 2:**
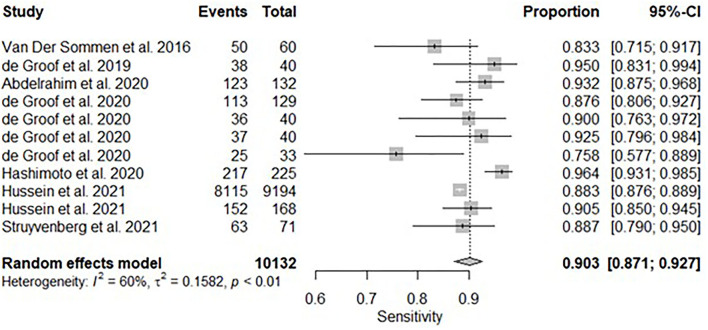
Forest plots of pooled sensitivities for all included studies of AI and the detection of early BE neoplasia.

**Figure 3:**
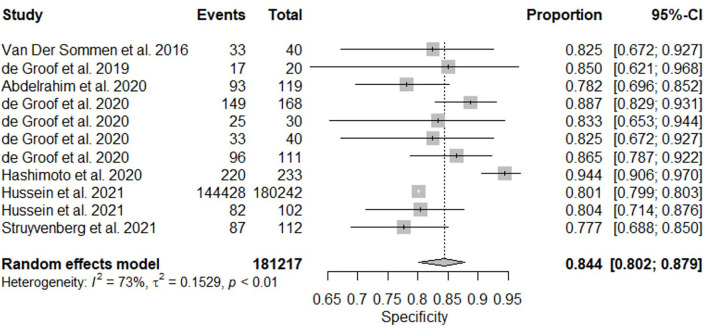
Forest plots of pooled specificities for all included studies of AI and the detection of early BE neoplasia.

**Figure 4:**
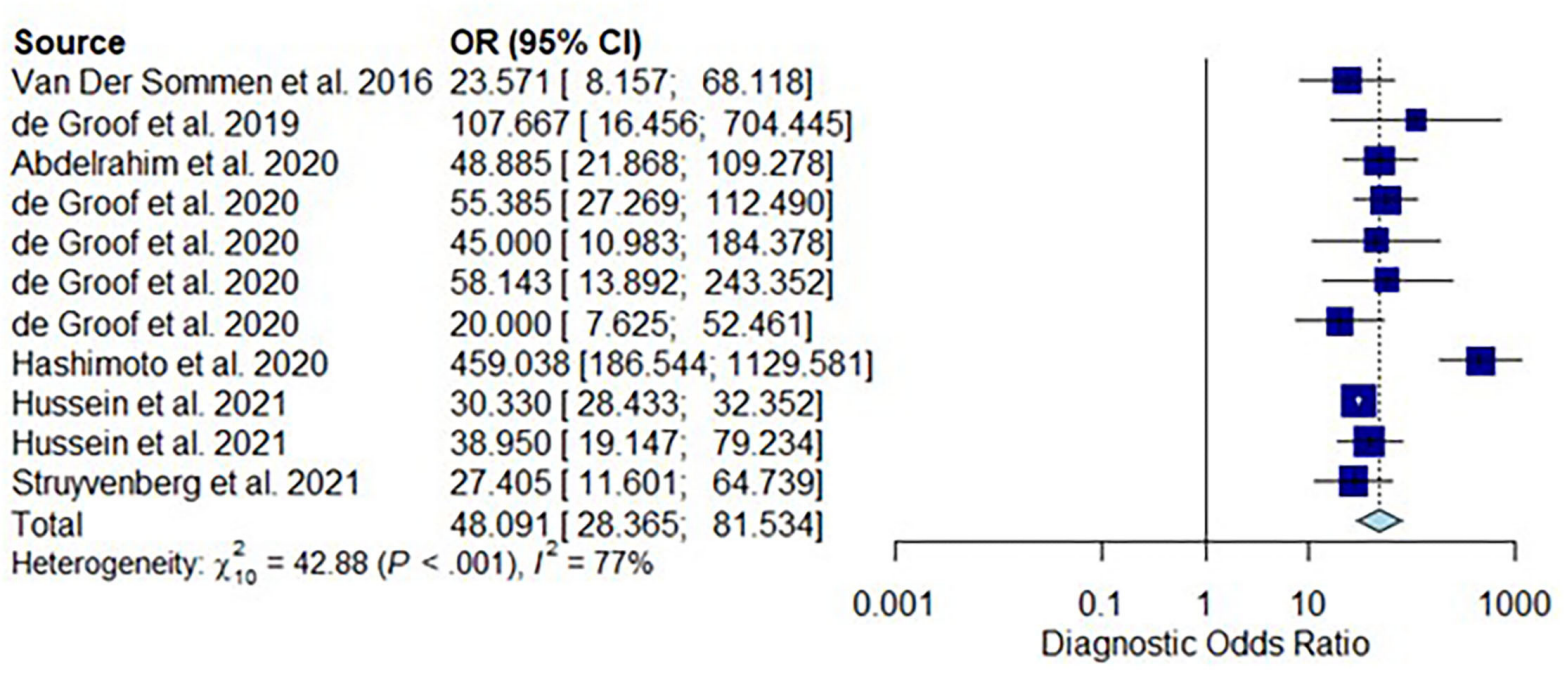
Forest plots of pooled diagnostic odds ratio for all included studies of AI and the detection of early BE neoplasia.

**Figure 5:**
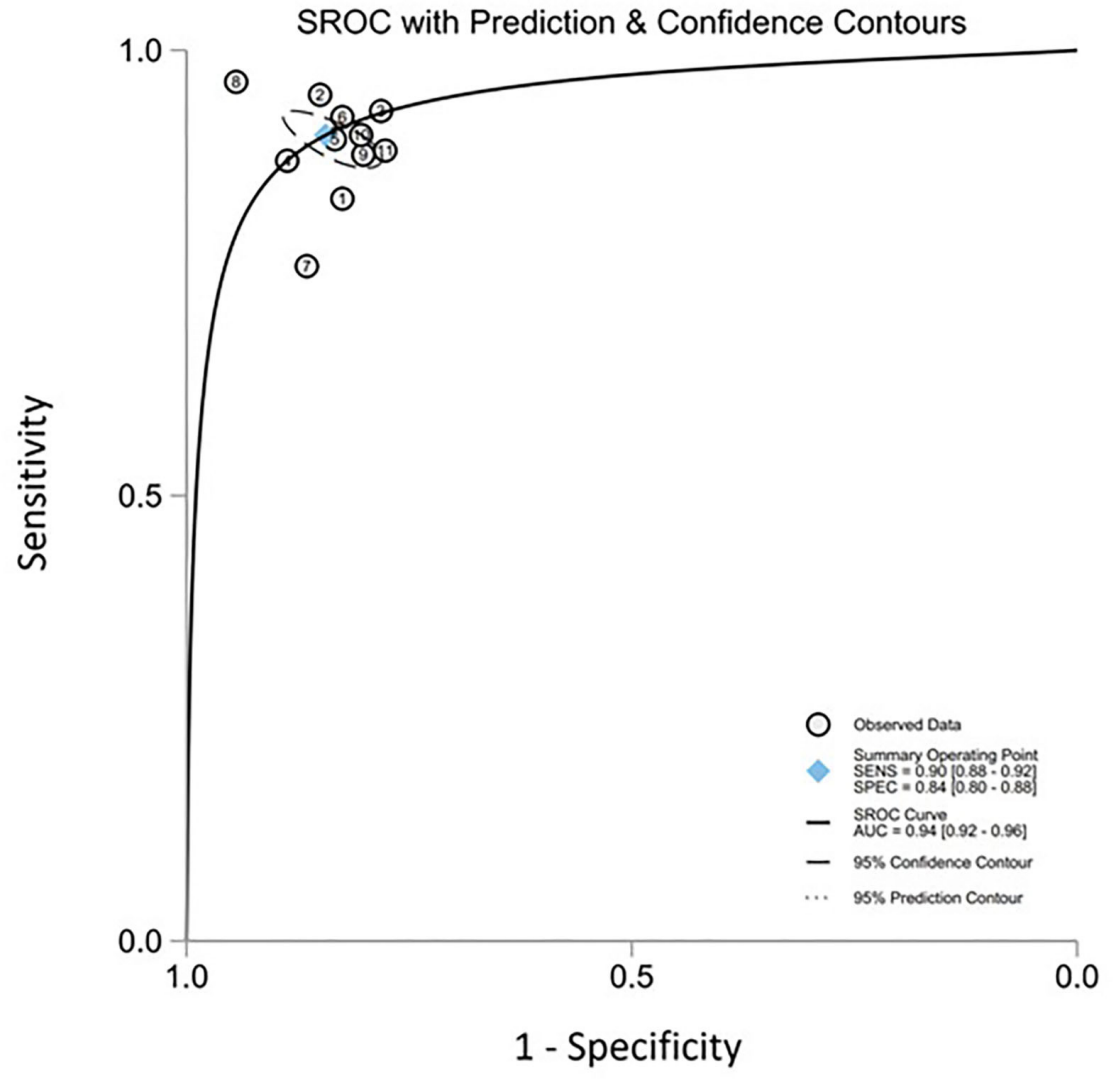
Summary of the receiver operating characteristics (SROC) curve of all included studies.

**Figure 6:**
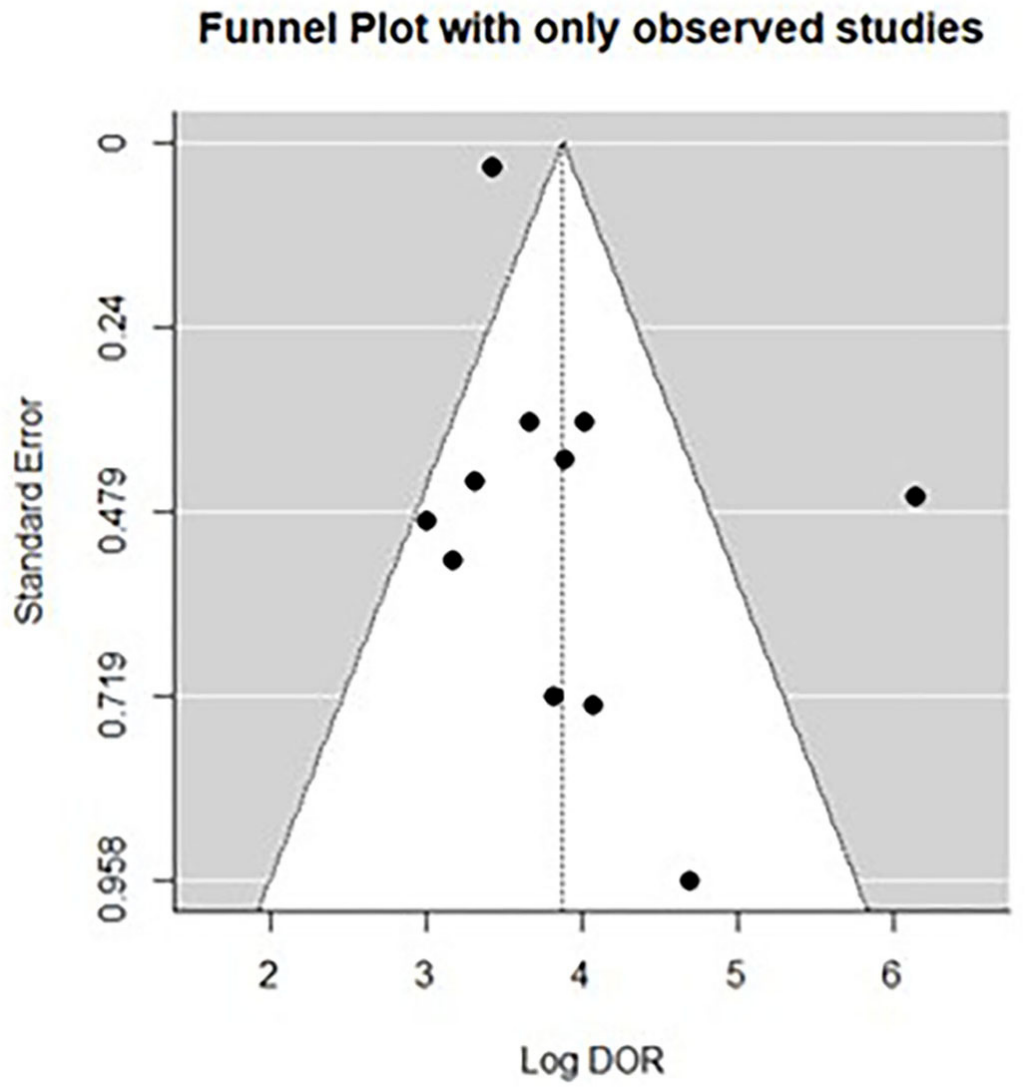
Funnel plots of all included studies.

**Figure 7:**
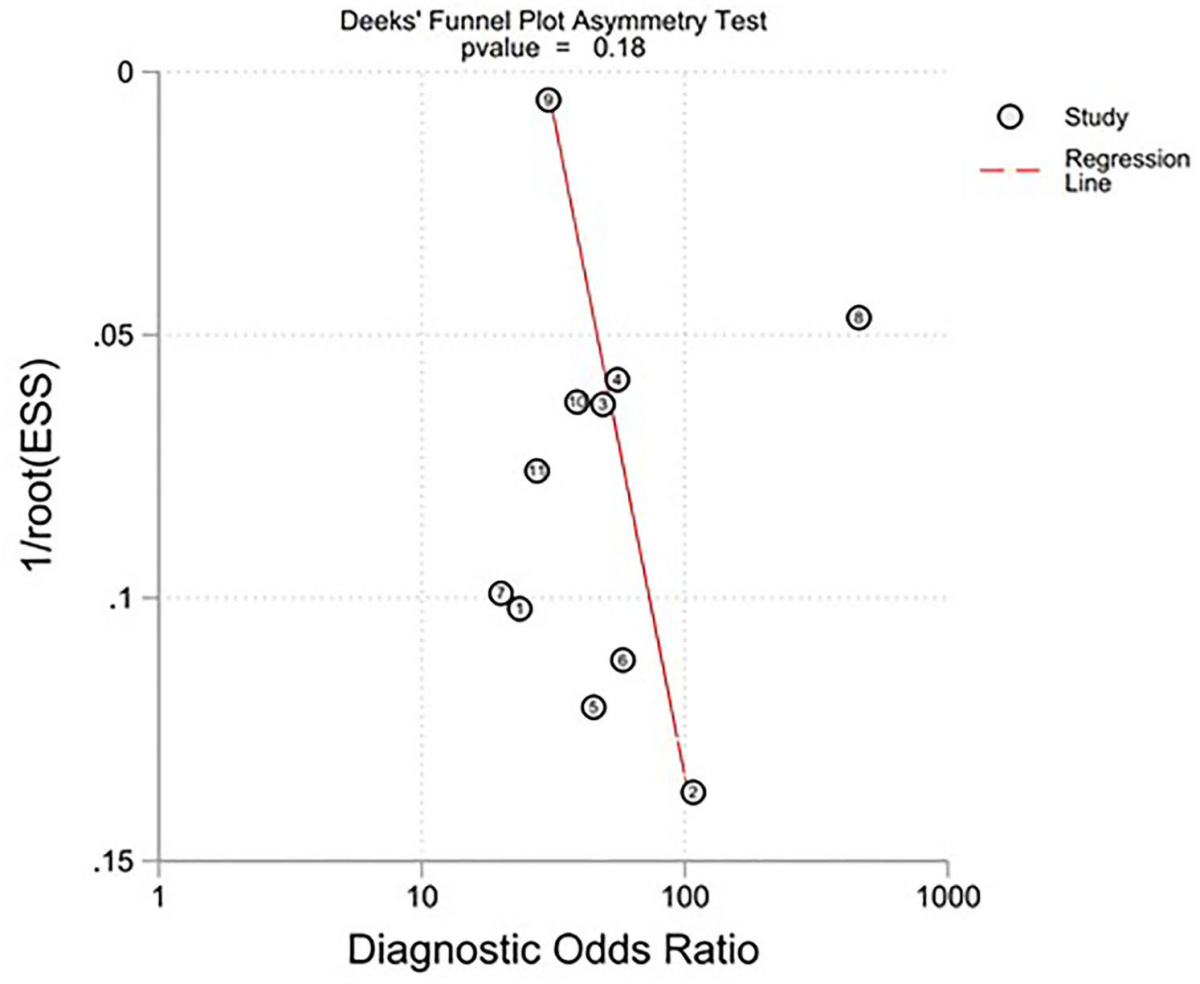
Deek's Funnel Plot asymmetry test of all included studies.

All twelve studies reported the sensitivity and specificity of their AI model at detecting early BE neoplasia. Eleven studies (16, 18–22, 25–27), including two studies which are sub-studies of de Groof et al. (20), of 191,278 endoscopic images or frames (for video-based studies) demonstrated that AI is very accurate at detecting early BE neoplasia with the respective pooled sensitivity, specificity and diagnostic odds ratio (DOR): 90.3% [95% CI: 87.1%−92.7%] (Figure 2), 84.4% [95% CI: 80.2–87.9%] (Figure 3) and 48.1 [95% CI: 28.4–81.5] (Figure 4). However, all the primary outcome analyses had significant heterogeneity (*I*^2^ of >50%, Tau^2^ of >0.1 and *p* < 0.05). Whilst there is significant heterogeneity amongst the included studies, the area under the summary of receiver operating characteristics curve was 0.94 [95% CI: 0.92–0.96] (Figure 5). Funnel plot assessment showed asymmetrical distribution, possibly due to underlying heterogeneity (Figure 6). However, Deek's test did not show statistically significant publication bias with a *p*-value of 0.18 (Figure 7).

### Subgroup Analysis–White Light Endoscopy

**Figure 8:**
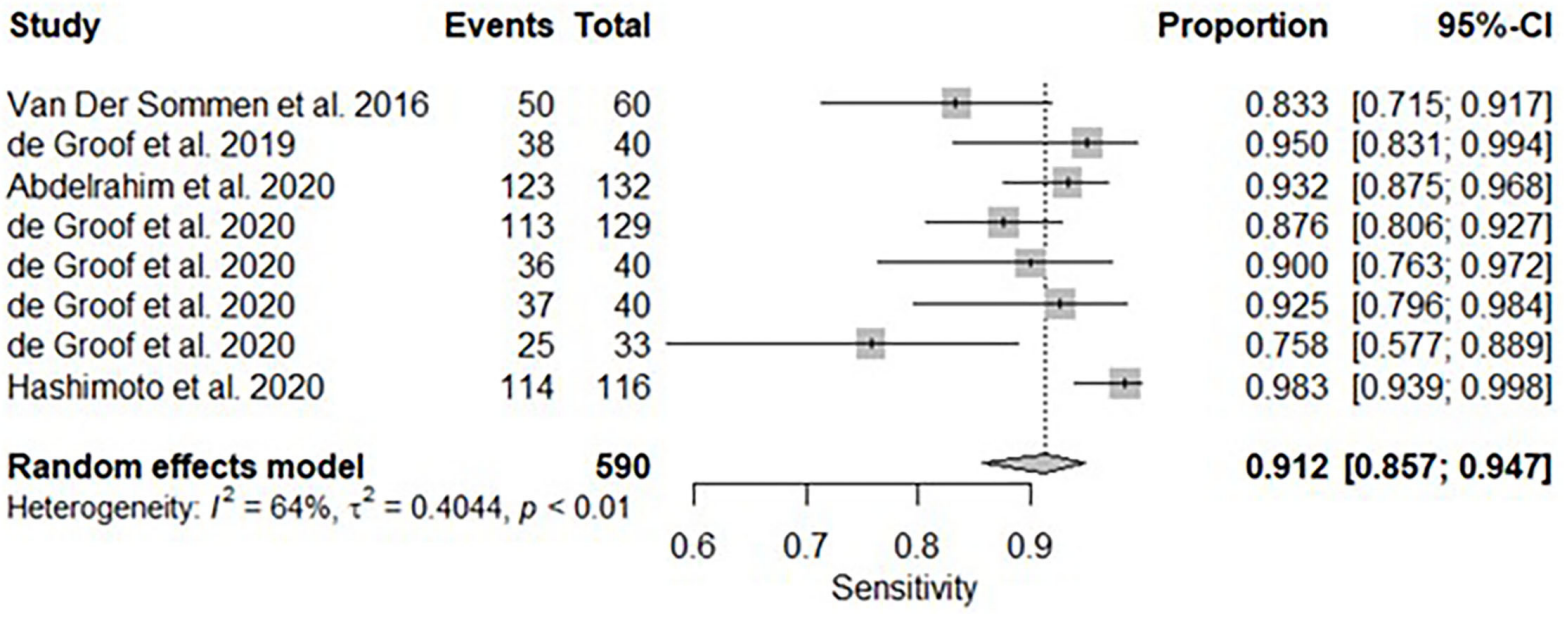
Forest plots of pooled sensitivities for included studies of AI and the detection of early BE neoplasia (White Light Endoscopy only) – Subgroup analysis.

**Figure 9:**
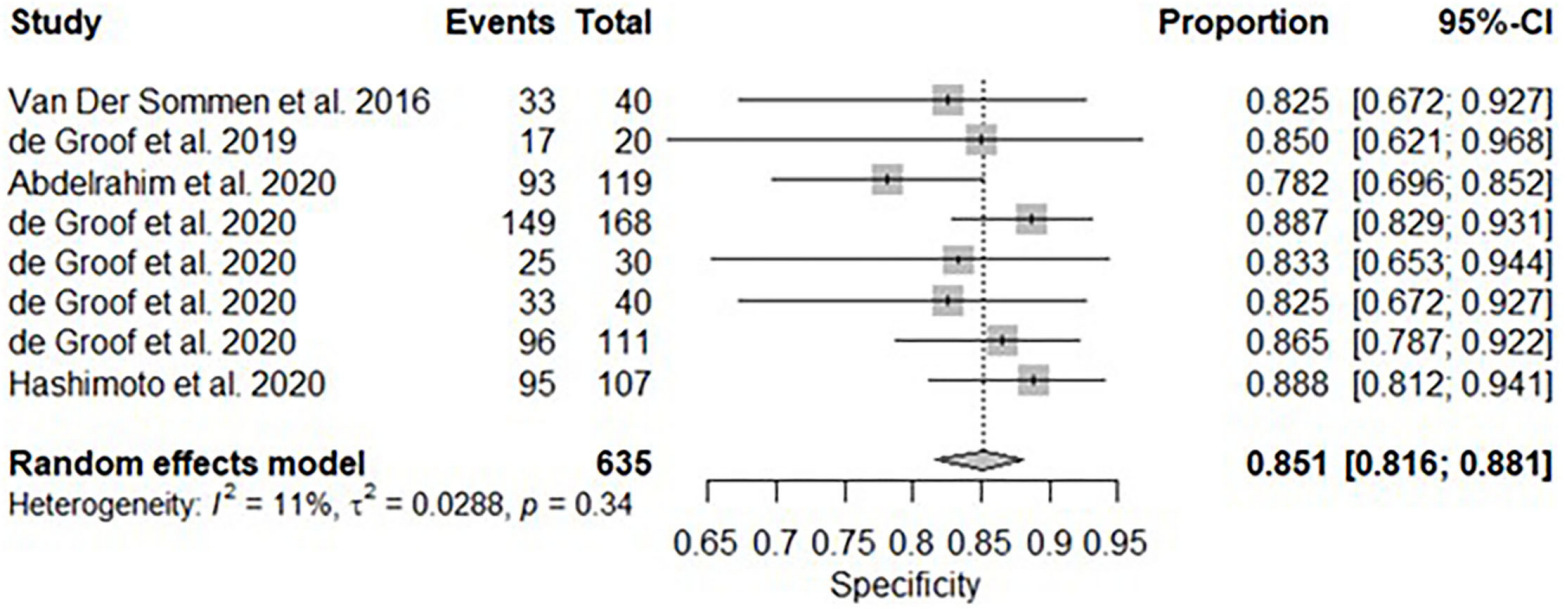
Forest plots of pooled specificities for included studies of AI and the detection of early BE neoplasia (White Light Endoscopy only) – Subgroup analysis.

A subgroup analysis was performed of six studies (16, 18–22), with White Light Endoscopy as the mode of imaging modality demonstrated similar pooled sensitivity and specificity: 91.2% [95% CI: 85.7–94.7%] (Figure 8) and 85.1% [95% CI: 81.6–88.1%] (Figure 9). There was significant heterogeneity for pooled sensitivity analysis (*I*^2^ = 64%, Tau^2^ = 0.40 and *p* < 0.01). Heterogeneity was not significant for pooled specificity analysis (*I*^2^ = 11%, Tau^2^ = 0.29 and *p* = 0.34).

## Discussion

This systematic review and meta-analysis of more than 500,000 images from 1,361 patients have consistently shown that AI can detect early Barrett's neoplasia accurately. An area under the SROC of 0.94 [95% CI: 0.92–0.96] was achieved with a pooled sensitivity of 90.3% (95 % CI: 87.1–92.7%) pooled specificity of 84.4% (95% CI: 80.2–87.9). Of note, three of the included studies (17, 23, 24) could not be included into this final analysis due to lack of required data but their respective sensitivity and specificity are represented in Table 1.

There is moderate inter-study heterogeneity noted in the included studies. Qualitative assessment of these studies showed multiple factors such as inclusion definitions of dysplastic Barret's lesions, different types of AI algorithm and imaging modality used. Whilst most included studies (17–27) used a CNN based AI algorithm, different pre-training datasets and CNN models were used. Similarly, different modalities such as white light endoscopy (WLE), narrow-band imaging (NBI) and “i-Scan” were used in various studies or combinations. All the above are very likely to contribute to the heterogeneity of our primary study outcome.

Subgroup analysis with WLE was consistent showing similar results with pooled sensitivity and specificity: 91.2% [95% CI: 85.7–94.7%] and 85.1% [95% CI: 81.6–88.1%]. Interestingly, in contrast to its pooled sensitivity analysis, pooled specificity analysis was homogenous. This finding could imply that whilst there is an array of AI models with different sensitivities, their respective specificities are limited by the macroscopic details of what optical white light endoscopy can achieve.

Compared to current published data, AI outperformed expert endoscopists in detecting early Barrett's neoplasia. In a benchmark assessment of 17 senior endoscopists conducted by de Groof et al., the following accuracy, sensitivity and specificity were achieved: 74.8% (95% CI: 72–77%), 76.5% (95% CI:72–81%), and 73.1% (95% CI:66–80%) (20). Accurate classification of BE lesions has significant clinical implications as it determines the duration of follow up, types of endoscopic resection required (for example, endoscopic mucosal resection vs. endoscopic submucosal dissection) or whether radiofrequency ablation is required (28). Ultimately, prompt management of early Barrett's neoplasia will likely improve patient mortality and quality of life.

However, without including BE lesions with low-grade dysplasia (LGD) in any of the included studies, it remains a challenge to determine whether AI could indeed detect all dysplastic lesions in patients with BE. Firstly, not all lesions of BE with LGD can be seen macroscopically. One study had demonstrated that of the 75 patients with a confirmed diagnosis of LGD, 52% were visible at the time of endoscopy in expert centers, with a significant contrast of only 12% detected at community centers (29). Moreover, the diagnosis of LGD is further limited by pathologists' poor inter-observer agreement of histopathological criteria (30). Whilst AI algorithm could theoretically improve the rates of detections of LGD, which may benefit community centers, it will be challenging to train an AI model without consistent “ground truth” of what is classified as LGD histologically by pathologists.

From an AI design perspective, instead of using pre-trained CNN models, de Groof et al. developed a model pre-trained using more than 500,000 endoscopic images of the gastrointestinal tract and subsequently refined with images of BE with or without dysplasia, verified by experts and histological reports (20). However, their results are similar to CNN models pre-trained conventionally based on ImageNet, an image database organized according to the WordNet hierarchy such as Hashimoto et al. (22).

There are various strengths to this meta-analysis. Firstly, there are more than 500,000 images from 1,361 patients in this review, which is currently the largest sample size in this patient cohort. Secondly, this meta-analysis is restricted to patients with BE and allowed us to ascertain the performance of AI at detecting early BE neoplasia. Thirdly, this systematic review presented the subtypes of CNN models used in each study which were not included in previously published reviews. Understanding differences in AI models is an essential aspect of interpreting the performance of each study, as AI models can be trained very differently, using different pre-training image databases and various refinement techniques.

Our meta analysis has some limitations. Firstly, most included studies were retrospective in nature. This can result in selection or information bias. Secondly, no studies included LGD due to issues as described in the above section. Thirdly, there was insufficient data to perform a subgroup analysis of the performance of AI vs. endoscopists at detecting early neoplastic BE. Fourthly, several studies (18, 24, 25) only included <100 unique patients in their training model. A concept of “over-fitting” may occur where the AI model becomes too attuned to the limited dataset on which it was trained and therefore loses its applicability to any other datasets. Lastly, there was significant inter-study heterogeneity in this meta-analysis which is likely multifactorial. Given the advancements of different AI techniques, it will be expected that future studies will likely be heterogeneous.

## Conclusion

In conclusion, this systematic review and meta-analysis provide updated evidence showing that AI is highly accurate at detecting early Barrett's neoplasia but validated for patients with BE lesions of least high-grade dysplasia and above. Our results support the need for more studies, including AI models to detect macroscopically visible low-grade dysplasia. In addition, well-designed prospective randomized controlled studies are needed to further explore if AI can indeed be effective both for experts and non-expert endoscopists.

## Data Availability Statement

The original contributions presented in the study are included in the article/Supplementary Material, further inquiries can be directed to the corresponding author/s.

## Author Contributions

JT performed an initial search and review of articles, data extraction, quality assessment of included papers, and prepared the manuscript. RM performed an initial search and review of articles, data extraction, and quality assessment of included papers. RW provided statistical advice and performed statistical analyses on extracted data. RS, MC, and GC provided supervision of the systematic review and meta-analysis. All authors critically reviewed, edited, and approved the final manuscript for submission.

## Conflict of Interest

The authors declare that the research was conducted in the absence of any commercial or financial relationships that could be construed as a potential conflict of interest.

## Publisher's Note

All claims expressed in this article are solely those of the authors and do not necessarily represent those of their affiliated organizations, or those of the publisher, the editors and the reviewers. Any product that may be evaluated in this article, or claim that may be made by its manufacturer, is not guaranteed or endorsed by the publisher.
