# Supplementary material for: Diagnostic Accuracy of Artificial Intelligence (AI) to Detect Early Neoplasia in Barrett's Esophagus: A Non-comparative Systematic Review and Meta-Analysis
Source: Front Med (Lausanne). 2022 Jun 22;9:890720. doi: 10.3389/fmed.2022.890720 (PMC9258946; doi:10.3389/fmed.2022.890720)
Supplement: Supplementary file 1 [file Table_1.DOCX]

Supplementary Table 1: Search strategies for Ovid Embase

| **#** | **Query** | **Results from 1 Feb 2022** |
| --- | --- | --- |
| 1 | (AI or Artificial Intelligence).mp. [mp=title, abstract, heading word, drug trade name, original title, device manufacturer, drug manufacturer, device trade name, keyword heading word, floating subheading word, candidate term word] | 83,129 |
| 2 | convolutional neural [network.mp](http://network.mp/). or artificial neural network/ or convolutional neural network/ or computer assisted diagnosis/ | 96,987 |
| 3 | (computer assisted detection or computer associated diagnosis).mp. [mp=title, abstract, heading word, drug trade name, original title, device manufacturer, drug manufacturer, device trade name, keyword heading word, floating subheading word, candidate term word] | 259 |
| 4 | deep [learning.mp](http://learning.mp/). or deep learning/ | 32,341 |
| 5 | 1 or 2 or 3 or 4 | 182,643 |
| 6 | dysplasia/ or esophagus carcinoma/ or Barrett esophagus/ or esophageal adenocarcinoma/ or adenocarcinoma/ or Barretts [dysplasia.mp](http://dysplasia.mp/). or esophagus dysplasia/ | 164,474 |
| 7 | oesophagus [tumour.mp](http://tumour.mp/). or esophagus tumor/ | 14,622 |
| 8 | 6 or 7 | 176,515 |
| 9 | 5 and 8 | 801 |

Supplementary Table 2: Search strategies for Medline Pubmed

| **#** | **Query** | **Results from 1 Feb 2022** |
| --- | --- | --- |
| 1 | "artificial intelligence"[MeSH Terms] OR (("convolute"[All Fields] OR "convoluted"[All Fields] OR "convolutes"[All Fields] OR "convoluting"[All Fields] OR "convolution"[All Fields] OR "convolutional"[All Fields] OR "convolutions"[All Fields] OR "convolutive"[All Fields]) AND "neural networks, computer"[MeSH Terms]) OR "diagnosis, computer assisted"[MeSH Terms] OR "deep learning"[MeSH Terms]  Translations  Artificial intelligence[MeSH Terms]: "artificial intelligence"[MeSH Terms]  Convolutional: "convolute"[All Fields] OR "convoluted"[All Fields] OR "convolutes"[All Fields] OR "convoluting"[All Fields] OR "convolution"[All Fields] OR "convolutional"[All Fields] OR "convolutions"[All Fields] OR "convolutive"[All Fields]  neural network[MeSH Terms]: "neural networks, computer"[MeSH Terms]  computer assisted diagnosis[MeSH Terms]: "diagnosis, computer-assisted"[MeSH Terms]  deep learning[MeSH Terms]: "deep learning"[MeSH Terms] | 207,196 |
| 2 | "barrett esophagus"[MeSH Terms] OR (("oesophagus"[All Fields] OR "esophagus"[MeSH Terms] OR "esophagus"[All Fields]) AND "adenocarcinoma"[MeSH Terms]) OR ("oseophagus"[All Fields] AND "neoplasms"[MeSH Terms]) OR "esophageal neoplasms"[MeSH Terms]  Translations  barrett's esophagus[MeSH Terms]: "barrett esophagus"[MeSH Terms]  oesophagus: "oesophagus"[All Fields] OR "esophagus"[MeSH Terms] OR "esophagus"[All Fields]  adenocarcinoma[MeSH Terms]: "adenocarcinoma"[MeSH Terms]  tumour[MeSH Terms]: "neoplasms"[MeSH Terms]  oesophageal cancer[MeSH Terms]: "esophageal neoplasms"[MeSH Terms] | 58,791 |
| 3 | 1 AND 2 | 393 |

Supplementary Table 3: Search strategies for Medline Pubmed

| **#** | **Query** | **Results from 1 Feb 2022** |
| --- | --- | --- |
| 1 | MeSH descriptor: [Esophageal Neoplasms] explode all trees | 1728 |
| 2 | MeSH descriptor: [Artificial Intelligence] explode all trees | 1261 |
| 3 | 1 AND 2 | 4 |
